# Early motherhood: a qualitative study exploring the experiences of African Australian teenage mothers in greater Melbourne, Australia

**DOI:** 10.1186/s12889-015-2215-2

**Published:** 2015-09-10

**Authors:** Mimmie Claudine Ngum Chi Watts, Pranee Liamputtong, Celia Mcmichael

**Affiliations:** College of Health and Biomedicine, Victoria University, PO Box 14428, Melbourne, Australia; School of Public Health & Human Biosciences, Faculty of Health Sciences, La Trobe University, Bundoora, Victoria Australia; School of Social Sciences and Communications, Faculty of Humanities and Social Sciences, La Trobe University, Bundoora, Victoria Australia

## Abstract

**Background:**

Motherhood is a significant and important aspect of life for many women around the globe. For women in communities where motherhood is highly desired, motherhood is considered crucial to the woman’s identity. Teenage motherhood, occurring at a critical developmental stage of teenagers’ lives, has been identified as having adverse social and health consequences. This research aimed to solicit the lived experiences of African Australian young refugee women who have experienced early motherhood in Australia.

**Methods:**

This qualitative research used in-depth interviews. The research methods and analysis were informed by intersectionality theory, phenomenology and a cultural competency framework. Sixteen African born refugee young women who had experienced teenage pregnancy and early motherhood in Greater Melbourne, Australia took part in this research. Interviews were audio recorded, transcribed and data analysed using thematic content analysis. Ethics approval for this research was granted by Victoria University Human Research Ethics committee.

**Results:**

Motherhood brings increased responsibilities, social recognition, and a sense of purpose for young mothers. Despite the positive aspects of motherhood, participants faced challenges that affected their lives. Most often, the challenges included coping with increased responsibilities following the birth of the baby, managing the competing demands of schooling, work and taking care of a baby in a site of settlement. The young mothers indicated they received good support from their mothers, siblings and close friends, but rarely from the father of their baby and the wider community. Participants felt that teenage mothers are frowned upon by their wider ethnic communities, which left them with feelings of shame and embarrassment, despite the personal perceived benefits of achieving motherhood.

**Conclusions:**

We propose that service providers and policy makers support the role of the young mothers’ own mother, sisters, their grandmothers and aunts following early motherhood. Such support from significant females will help facilitate young mothers’ re-engagement with education, work and other aspects of life. For young migrant mothers, this is particularly important in order to facilitate settlement in a new country and reduce the risk of subsequent mistimed pregnancies. Service providers need to expand their knowledge and awareness of the specific needs of refugee teen mothers living in ‘new settings’.

## Background

Globally, teenage pregnancy remains a public health concern. Worldwide, sixteen million girls give birth during adolescence annually with an estimated three million having unsafe abortions. Most adolescent pregnancies occur in developing countries, and teenagers living in socio-economically disadvantaged settings in developed countries are at higher risk of teenage pregnancy as compared to the broader population [1]. The adolescent period is considered a critical time in the young person’s life. Initiation of sexual activities, and for many a marriage, occur during this period. The early onset of sexual intercourse and menarche and the delay in marriage means the period of adolescent is now longer than ever, which increases the risk of unplanned pregnancy and early motherhood. During the teenage years, young people who are faced with early motherhood may experience conflict between their new position as mothers and their adolescent needs [1, 2]. The experiences of early motherhood are contextual, influenced by culture and the society within which the teenager/woman lives [1–6].

In Australia, teenage birth rates fell from 22.1 live births per thousand women in 1992 to just over 15.5 births per 1000 women in 2010 [7]. Teenagers living in low socioeconomic areas have higher birth rates [8]. The consequences of early pregnancy and births to teenagers - including events leading up to these pregnancies - have been highlighted in the research literature [9]. Teenagers most at risk of unplanned pregnancies are those from low socio-economic status, families with a history of teenage pregnancies, those who have experienced abuse, and those without a father figure. Disconnections from school or leaving school early are also risk factors for and consequences of early pregnancy and birth. Some of the health risks to the baby include still birth, low birth weight, risk of dying in the first few months of life, and these risks increase with younger maternal age. For the mother, risks of fistula and maternal death, particularly in poor settings, are real. Some of the social problems include school dropout which leads to reduced educational opportunity and low skills acquisition [10]. However, a growing body of research has questioned the evidence that teen childbearing largely has negative consequences for teen mothers and their babies, and have highlighted the importance of understanding the views and experiences of teen parents [11, 12]. Regardless, after the birth of the baby, mothering and motherhood become a reality.

Motherhood is an important part of many women’s lives, particularly in societies where traditional gender roles persist. In many African societies, motherhood is central to the social and cultural system [13]. Motherhood and childbearing among Sub-Saharan African women is regarded as a normal duty within a woman’s life [14–16]. In many parts of Africa, motherhood is seen as an essential role, with family and social life orientated towards children with early onset of childbearing and large families preferred [10]. From an early age, there is a positive orientation towards motherhood [1, 13]. The importance of motherhood may continue post-migration and settlement [14, 17]. Yet motherhood in the context of migration is often substantially different: young immigrant women experience the dual transitions of becoming a mother while also adjusting to everyday life in a site of settlement, often without extended networks of social support [6, 18].

Early positive orientation towards motherhood has been associated with teenage pregnancy (TP) [4, 19]. Girls are made to feel that motherhood is a prerogative in their lives as women and central to female gender roles [4]. Australia is home to over 280,000 persons with African ancestry [20]. However, experiences of teen pregnancy and early motherhood among African Australians following migration are under-examined and inadequately understood. This paper aims to highlight the experiences and challenges of African Australian teenage mothers who are living in Greater Melbourne. It discusses their experiences of teenage motherhood, and critically examines how young teenage mothers - who are mainly single - navigate early motherhood.

In this paper, we use the terms teenager, young woman/woman and adolescent. Teenagers (13–19 years of age) are a subset of the adolescent period (10–19 years of age) [16]. All 16 young women who participated in this study were teenagers at the time of their first pregnancy; at the time of interview not all participants were still teenagers, but all were still young women below the age of 30 years.

### Theoretical framework

In this paper, we situate our finding within intersectionality theory which recognises the multiple intersections in a woman’s life, including race, gender, skin tone, accent, education level, migration status, language and other life situations [21–23]. Intersectionality theory considers the multiple dimensions within which teenagers exist, including gender, age, developmental stage, socioeconomic status, ethnicity, minority group status and migration experience (e.g. refugee). For the teenagers and young women who participated in this research, the above dimensions were a central part of their identities. Thus, intersectionality theory goes beyond the boundaries of race and gender to include other social categories such as migration status, religion, sexual orientation, educational attainment, language and many other categories that play and/or influence the individual’s life situation. Intersectionality theorists argue that to be able to understand the world of minority women, it is critical to move beyond the boundaries of gender and race. For Sub-Saharan African (SSA) women, culture, marriage, and child bearing remain important. Marriage and childbearing almost often define a woman’s position within the family and her community [13, 14]. Using the single axes of either race or gender will not present a complete picture of the individual’s experiences and cannot fully answer questions about the woman’s existence [21, 22]. To understand the position and the experiences of the teenage mothers in this study, their cultural heritage, the community associations and the lives or journeys they have experienced should be considered.

Intersectionality theory uses a multiple axes approach: it considers multiple complexities and dimensions, and the many identities that an individual woman may possess [23]. Each aspect of a person’s identity influences their decision making. For African teenage mothers who are at the same time refugees, from low socioeconomic background and with low levels of education, these multiple identities need to be understood when examining experiences of early pregnancy and early motherhood among this cohort of migrants. While intersectionality has been critiqued as being too open [24], we posit that as a framework it captures the nuances and differences that are central to individual lives [21, 22], including the young women in our study.

## Methods

Qualitative research is widely used in the health sciences and is regarded as the most appropriate method when exploring people’s life experiences or phenomena that are sensitive or socially complex [25, 26]. This study utilised in-depth interviewing methods, and the study drew upon both phenomenology and cultural competence frameworks to inform the research methods and analytical approach. Phenomenology was particularly suited for this study as it is concerned with the study of human existence and how humans understand and perceive their own behaviours [27]. Phenomenology allows the researcher to uncover hidden aspects of people’s lives that would not emerge during ‘normal’ conversations, or that people would not typically reveal to people outside their own social or cultural circles ([27, 26]).

In-depth interviews were chosen as the primary data collection method as their structured nature allows the interviewee to ‘tell their story in the deepest and richest way possible during the interview process’ ([28] p. 388). Participants were eligible to participate in the study if they were: (i) of African descent; (ii) had migrated to Australia under the Australian Humanitarian scheme, or were sponsored by someone who had migrated under the humanitarian scheme; and, (iii) had experienced teenage pregnancy (TP) or early motherhood. Participants were included from different African ethnic and cultural groups, different socio-economic situations, and from different settings within greater Melbourne.

Purposive sampling was used to reach this hard to reach population as it allowed the researcher to interview those who had experienced teenage pregnancy. Initially, invitations were sent out to potential participants through formal (church notice board) and informal (friends and community members) networks. Potential participants were invited to contact the researcher and set up a convenient interview time. This method was not successful in recruiting African women who had experienced TP. Another researcher in the UK has also reported very low response rates when recruiting black African families using information flyers and the internet, and subsequently used snowball recruitment techniques via formal and informal social networks [29]. Accordingly, in this study snowballing methods were ultimately used to identify potential participants who met the eligibility criteria. People who heard about and were interested in the research referred potential information-rich participants [26]. Potential participants were provided with a plain English language statement about the research. It was only after this process that interviews were set up with participants. This allowed the participants opportunity to consent to participate, or to opt out or cancel the interview if they did not want to proceed.

In-depth interviews were conducted with sixteen African Australian teenage mothers, or women who had experienced teenage pregnancy, and who had a refugee background (see Table 1). Pseudonyms were assigned to all participants to ensure confidentiality [25]. Interviews were digitally recorded and transcribed verbatim. The data were entered into NVivo (qualitative data analysis program) and analysed using thematic analysis [26]. Data were read for understanding several times. An inductive analysis and exploratory approach was applied during this process. Coding, sorting and organising data are an integral part of thematic analysis [30]. The data were searched systematically for re-occurring words, which later became code words: these code words were then grouped to form themes. The NVivo software was used in conjunction with manual coding during the data analysis to help with the management of the data.

**Table 1:** Demography of 16 African Australian Teenage Mothers with Teenage Pregnancy Experience

Employment status	Gender	Current age	Country of Birth (length of stay)	Transit country (countries)	Approximate length of stay in Australia	Main languages spoken at home
Unemployed	F	20	Ethiopia	Egypt	5 years	Arabic, Dinka
Unemployed	F	21	Sudan	Uganda	4 years	Dinka, English
Unemployed	F	19	Liberia (2 months)	Guinea 10 years, Kenya 5 years)	4 years	Gio, Eglish
Employed (casual)	F	24	Sudan	Egypt	8 years	Dinka, Arabic
Unemployed	F	27	Liberia	Not stated	9 years	French (other)
Unemployed	F	19	Sudan (North)	Egypt (7 years)	Not stated	Arabic, Dinka
Unemployed	F	18	Sudan (2 months)	Kenya (2 years)	16 years	English (a bit of Dinka)
Unemployed	F	30	Sudan	Egypt	Not stated	Arabic, Dinka
Unemployed	F	22	Sudan	Egypt	6 years	Nuer, Arabic
Unemployed	F	20	Burundi	Tanzania (8 years)	5 years	Kirundi, Kiswhahili
Unemployed	F	17	Sierra Leone	Not stated	2 years ago	Timinie, Creole
Unemployed	F	17	Sudan (7 years)	Uganda (6 years)	3 years	English (little Dinka)
Unemployed	F	Early twenties	Sudan	Egypt	7 years	Arabic, Moro
Unemployed	F	28	Sudan	Egypt	Not provided. Had lengthy stays in refugee camp prior to arrival in Australia	Arabic, Dinka
Unemployed	F	17	Liberia 5.5 yrs	Guinea, Liberia, Sierra Leone	4 years	Gio, Madingo, Mana, Berle, French, English
Unemployed	F	18	Sudan	Not stated	2 years	Dinka

Ethics approval for this research was granted by the Victoria University Human Research Ethics Committee. Data collection took place between February 2010 and August 2011.

## Results

### Characteristics of the teenage mothers

The sixteen young women in this study had all migrated to Australia from Sub-Saharan Africa via Australia’s humanitarian program and fourteen had subsequently experienced teenage pregnancy. One arrived pregnant but was unaware of her pregnancy and one had both pregnancies overseas (in transit country) prior to arrival. At the time of interview, participants ranged in age from 17 to 30 years (one participant did not provide her age but was in her late teens to early twenties). All women were Conclusionsunemployed except one who had a casual job at a supermarket. Of the sixteen participants, ten were from Sudan, three from Liberia, and one each from Burundi, Ethiopia and Sierra Leone. All women, but for two, had lived in a transit country following flight from their country of origin, with some living in so-called transit situations for up to seven years. All participants spoke at least two languages and/or dialects. All women had a religious affiliation: fifteen were Christian and one a Muslim.

### Cultural influences

Regardless of whether the pregnancy was planned or unplanned, all the teen mothers in our study decided to proceed with their pregnancy. A few indicated that knowledge of others’ experiences of crude abortions in refugee camps was a deterrent to aborting the baby. Cultural and religious attitudes and practices further influenced the decision to carry the baby to term, despite the challenges of single/teenage motherhood. Chelsea, a young Muslim woman, discusses her fear of abortion, the fate that awaits a woman if she dies due to an abortion, and the implications of abortion for the family:

*In the camp, one girl, she was pregnant and she was a Muslim girl and she got pregnant by a Christian boy and then the Christian boy denied the pregnancy and then she went and drank something to get an abortion and then she died. So that one make many people scared in the camp. Everyone would say, “I’m not going to do abortion anymore”. And then when she died, like a Muslim, you do abortion no one will touch you. They have to get someone to take you away; they (Muslim) can’t even bury you. No one will come next to you (the body). So it was so sad. Her family members crying, “There’s no one to bury her” from the Muslim community. (Chelsea)*

However, women also spoke of more positive reasons for proceeding with pregnancy. Kayla, for example, was 19 years at the time of interview and was pregnant. She said ‘*I thought if I got pregnant our life would be better . . . that’s why in my second relationship I said, “I just want to get pregnant. I don’t care from whom but I just want a baby”.* The ‘cultural socialization’ for some of these teen mothers was that motherhood was just part of life whether planned or unplanned.

### Becoming a mother

Among these young women, becoming a mother was largely a positive experience, despite the associated challenges. They were generally happy to have a baby of their own and felt that their lives had changed for the better since becoming a mother, even when everyday life was difficult. Motherhood was perceived to be a connection and an avenue for their parents to accept a partner they would otherwise not accept. According to Candida:

*I thought if I get pregnant and have a baby together with him, mum will not be able to do anything about it and we will be together because of the baby.*

Motherhood, however, also brought some mixed feelings and experiences. Pregnancy at any age can be physically exhausting, and for young women who are often ‘alone’ or with little support, and physiologically and emotionally still developing, pregnancy can be challenging. Having someone to lean on irrespective of their age can bring feelings of joy to these young women. For Alimatou, a mother to a two year-old boy and expecting a second baby, support was provided by her son. Below Alimatou shares her joys and sorrows of motherhood as a pregnant young mother:

*In a way, sometimes it’s good, sometimes not good … sometimes when something happens and we are in the apartment he says, ‘Sorry, Mum.’ Then when I’m tired, like when I’m sleeping, I’ll send him and he’ll go and bring something so that I don’t have to go and bring them.* (*Alimatou)*

For some young women, motherhood brought with it a sense of maturity, elevated responsibility and purpose. They began to regard themselves as adult and more mature. Becoming a mother meant they had to behave like responsible adults. Motherhood offered them an immediate family structure, and gave them a person they could truly love. For some, it brought an increased sense of self-worth. For Francisca, motherhood did not only bring joy, she felt more grown up and ‘ahead’ of her peers:

*Now I’m like a woman. I’m sort of a girl and a woman. I’m an older girl, not young girls that are getting new stuff. I’m not one of them … Because I had a baby and comparing to the girls who haven’t had a baby.* (*Francisca)*

While there was a sense of purpose and maturity that came with motherhood, these mothers acknowledged the many difficulties they faced as young mothers.

### Challenges of motherhood

Despite the positive elements of motherhood, challenges emerged that affected various aspects of the young women’s life. Most often, these challenges stemmed from coping with the responsibilities of looking after a baby and young child, particularly when also attending school and/or seeking employment:

*Yeah, is very hard to take care of the kids and still go to school or look for job, and you don’t have someone to look after you and your baby. It’s very hard. (Daniela)*

Some women felt regret in relation to having a baby while still at school, particularly when they were unable to complete their school education which then led to difficulties in finding work. This difficulty was noted particularly among those with limited social support networks, as they had no one to help at home or lend a hand in the absence of biological parents following migration:

*It is not easy to have a baby. It’s very hard, it is better to go to school and get a job first. Once the baby comes, it is very hard, you can’t do anything, especially when you do not have someone to help you it is very hard. (Ayuba)*

Those with an older child found the tasks of motherhood even more demanding. Meeting the needs of the older child and a new-born, continuing their own education, and socialising with their friends were reportedly very difficult for these teen mothers. Feelings of exhaustion were not uncommon, making it difficult for the young mother to re-engage with or enter the work force, or to pursue training or education. These challenges were more evident when the young mother was expecting another child, often with limited resources and support. Below Jessica, aged 17, with a young daughter and expecting her second child spoke of how hard everyday life had become. Jessica compared her current situation with the time she only had Rosy to parent. Jessica, who did not have her biological parents in Australia, highlighted the daily life challenges she faced coupled with the physiological changes expecting mothers have to deal with:

*It’s very difficult. Before it was easy for me before I just had Rosy. Because she couldn’t walk, I couldn’t take her to the shop, I can’t go to school and I can’t do all my stuff. But now I don’t do anything because if I take her to the child-care I come back home and I’m just tired. I think when the baby is born it will be more difficult because I don’t have a car because he [her boyfriend] took my car and I don’t even have a car and that’s why everything is getting more difficult. It will be very hard for me to go back to school now. Everything is not going to be easy like it used to be. But I am happy with my kids. (Jessica)*

### Post-partum social support for the teenager

Experiences of stress and regret that came with teen motherhood were associated with inadequate social support before and after the baby’s birth. There was a widespread sense of loss of social life and inadequate social support. Among these women with African backgrounds, the lack of social support in a site of settlement emerged as a common difficulty that had an impact upon their experiences of early motherhood, everyday life, and plans for their future. However, some support was available to assist them to meet the demands and challenges of early motherhood.

#### Family support

The challenge that many migrants face in sites of settlement is the lack of extended family, social and cultural networks [6, 14]. Family and friends are considered a source of support, and for teenagers this is significant in how they will reintegrate with education, employment and social life. Some of the young mothers in this study migrated alone, some with extended family member and family friends. Most young mothers received some support from their parents and guardians (i.e. people who sponsored the young women to come to Australia, or who were caring for them when the pregnancy occurred). The extent of support that the women received from these people depended on the relationship they had before the pregnancy and birth of the baby. Where the relationship between parents or guardians and teens had been good, they were likely to receive support. Teenagers who lived with at least one biological parent or a first degree relative received the most help and support with the baby as compared to those who did not live with their parents or relatives. The young mothers who lived with guardians said they would have had better support if their biological mothers were present:

*Is not good thing [to be pregnant out of wedlock] but she’s [uncle’s wife] not good since I get pregnant. She kicked me out of house. Now she doesn’t talk to me, even if she finds me in the street she doesn’t say hi to me. It’s not good. So it’s better to have your mum. Even if your mum is angry with you it’s not going to be like this. She’s going to calm down a little. (Daniella)*

Mothers were reportedly at the fore when it came to supporting the teen mothers, even if they had been unhappy or disappointed about their daughter’s pregnancy. Those participants who had the support of their mothers indicated that their mothers had a sense of responsibility towards them. Their own mothers’ acts of love towards the young mothers and their baby were highly valued and acknowledged. The support of participants’ mothers and families was evident in some interviews. The interview with Chelsea took place at her home, and Chelsea’s mother had the baby on her back (as is the practice for African mothers) while Chelsea went about her interview and other duties. When the baby cried, her sister who was about 12 years-old at the time, carried the baby and cuddled her. It was evident that Chelsea had support from both her mother and sibling. Chelsea said her sister had learnt from her not to have a baby while still at school or out of wedlock, although she continued to help her with the baby. In other interviews, female siblings were evidently helpful and supportive of their sister and her baby. Several young mothers received help from their sisters in taking care of the baby so they could attend to school work or go out and socialise.

The level of support received by young mothers substantially influenced their intentions and capacity to re-engage with education and work. Teenagers who received more support from their family, especially from their mothers, were more likely to return or want to return to school. Chelsea, for example, had the support of her mother and went back to school when her baby was aged four months:

*[My mum] asked me ‘Are you going to keep the baby?’ and I was like, ‘Yes.’ My mum was upset because of my schooling and stuff and then I talked to my mum and we had a fight for some time … so she says I have to go to school . . . As soon as February, when school started I went back to school, because I wanted. I wanted to do something for myself in the future that’s good. I want to become a nurse. (Chelsea)*

Those young women who had their fathers in Australia did not feel strongly about getting their support. For most of these teenagers, their fathers were partially or completely absent from their own lives, which often brought feelings of loss. Almost all teenagers came from single parent homes or their fathers were reportedly in Africa or elsewhere, often married to other wives:

*Dad, he’s in the USA. It’s very strange, we’re trying to be close to him but his mind is always somewhere else. I’m not sure if he wants to have children and a family even at his age. He left when my brother was about one year and my younger brother is 15, turning 16, so it’s like we haven’t seen him for 15 years. It’s feeling like I’m sad. (Kayla)*

Support was not only lacking from absent biological fathers; step-fathers who were present were said to provide limited support for the step-daughters and their children. Bikutsi, who had two miscarriages, felt unsupported and unloved by the ‘fathers’ in her life, including her step-father and biological father. Indeed, she attributed some of the struggles in her own life (and those of her mother) to the inadequate support of the fathers.

#### Support from friends

Participants received mixed levels of support from friends, and the level of support largely depended on the type of friendship before the pregnancy. Loyal friends were said to support the mothers during the pregnancy and after the baby’s birth:

*Some [friends] are very close, like this one who is here now, she visits daily. Even before I had the baby she encourages me. The others are now very distant from me. I think because I was pregnant. (Veronica)*

During the interview with Veronica, her friend was present and helped out with the baby. They seemed very close and, according to Veronica, becoming pregnant strengthened the relationship between the two friends. This supportive scenario between friends was not the same for others, who felt isolated, sometimes because their friends were young mothers themselves:

*I have one best friend and she has a baby as well, how can she support me? She has to look after her baby so she didn’t have time to support me. So I decided to keep to myself. (Chelsea)*

For some young mothers, long travel distances and the inability to commute easily was a barrier to getting support from friends:

*Yeah I have one [friend] but she lives very far. She lives in the other side of the city. I do not drive, so it’s very hard to get there. (Ayuba)*

#### Support from the baby’s father

The fathers of the babies were often absent during and after the pregnancy. For the few fathers who were around, the amount of support provided - financial, emotional or physical - to the teenage mother and the baby was generally described as inadequate. When present, the a few fathers were reportedly inclined to try to access the social security benefits available to the mother rather than support her with the child:

*He would pretend that he loved me, but he didn’t love me and he didn’t love my child. It’s very hard to find a good person because when they know you have a baby, they don’t care about you. They love to come around if you have money and ask if you have money and then they just go away with the money. If they know you have a kid, it sends them packing. (Stephanie)*

For some teen mothers, a perceived lack of support from the father was due to the fact that the relationship had broken down before the baby’s birth. As Veronica said:

*We are not getting married, yes [we are] in a relationship; no, not serious, just between the baby … Because I don’t love the boy … because my mind is not to love the boy … My heart is not there … I am young and I want to continue my education.*

#### Support from the wider community

In Melbourne, members of the wider African community generally frown upon unmarried teenage mothers, leaving the teenagers with feelings of shame and embarrassment. Young mothers are perceived to set bad examples to other teenagers, and give a bad reputation to the community and their families. Hence, teenage mothers were perceived to be bad role models for other younger girls. In accordance with this perception, the lack of support given to these mothers by members of their community was evident. None of the participants in this research said they had received support for the baby via their community. Community attitudes towards them left with feelings of embarrassment. Veronica noted:

*I was embarrassed; I was embarrassed with everyone [in the community]. My mother was angry with me, because I could not go to school. She felt bad, because I am pregnant in street [unmarried] and it is a big problem in the community.*

According to the participants, an African family is situated within the wider community, and they share in their children’s feelings of shame and embarrassment that emerge through wider community disapproval. A daughter’s teen pregnancy out of marriage reflects badly on the parents, and places their parental responsibilities/duties in question by the wider community. Interdependency post migration remained an anchor point for many African migrants and their families. Thus any community perceptions and attitudes towards individuals were taken seriously. Those young mothers without significant social bonds expressed feelings of exclusion and rejection. Francisca who moved to Melbourne from another city without sharing her pregnancy status with anyone recounted:

*Because I haven’t got any friends that connect with me, like really best friend, I can’t just talk to all the girls that sit with me and I don’t know really well and haven’t met them. (Francisca)*

Stephanie shared similar feelings of isolation and a lack of ties with her ‘friends’:

*If I go and tell my friend something about me, maybe they communicate it to other people I’m better keeping it to myself. (Stephanie)*

Friendships were limited for many of these young women because of their migration statuses and interrupted lives. Together with other barriers, integration into the wider community was difficult for them; becoming pregnant and negative community perceptions of teen mothers increased young women’s risk of isolation.

## Discussion and conclusion

The study provides insight into young African migrant women’s experiences of teen pregnancy and early motherhood, with particular focus here on their experiences of social support. The paper is based on qualitative research – i.e. in-depth interviews with sixteen African Australian women with refugee backgrounds who experienced teen pregnancy - and the findings cannot be taken as representative of the experiences of all refugee and African young women in Melbourne, Australia. However, the paper does provide insight into the intersecting identities that shape teen pregnancy and early motherhood in a site of settlement.

For African Australians with refugee background living in Melbourne, teenage motherhood brings both joy and regret [14]. This finding supports the increasing body of research that indicates teen childbearing should not be viewed solely through a ‘risk prevention’ lens that emphasises negative consequences for teen mothers and their babies [11, 12]. Our research indicates that motherhood brought happiness for many of the young women and an increased sense of meaning, despite the associated challenges of early parenting [14]. There was an associated sense of maturity and responsibility. Many young mothers developed a personal sense of stability, identity, purpose and responsibility following early motherhood, a finding similar to other studies of teenage pregnancy and parenthood (see [1, 6, 14]). The respect awarded mothers in Africa may be seen as a reason for this strong sense of identity and purpose following motherhood [1]. In this study, positive experiences of motherhood were associated with good social support which contributed to feelings of acceptance and optimism for teenage mums (see also [1]). For some, teen pregnancy and early motherhood brought them closer to their families (particularly mothers and female siblings) and they valued having a child whom they loved and who loved them back [1, 4]. Other studies have reported that pregnancy and motherhood can strengthen relationships and seal the woman’s place in a relationship, marriage and within the community [1, 6, 14]. Indeed in some societies, particularly when political instability is common, women become bearers of nationhood [3]. Thus, society, culture and the context within which motherhood occurs shapes and influences the motherhood experiences.

Nonetheless, in this study, all the young mothers indicated that pregnancy and early parenthood had come at a time when they were also confronting the ongoing challenges of settlement in Australia as well as the transitions and challenges of adolescence and early adulthood. Our findings demonstrate the conflicting role of motherhood to the self despite inherent benefits to the self, family and broader society [6, 14]. Other studies have documented the conflicting experiences (joy and challenges) of motherhood, particularly among adolescent mothers who experience tensions between their needs as both mothers and as adolescents [2]. For participants in this study, they negotiated the competing demands and challenges of pregnancy and parenthood as well as adolescence and early adulthood, while also confronting settlement in a new country which brings its own challenges in relation to housing, language acquisition, education, social connections and workforce participation. Becoming a young mother in a new country is therefore complex, and theoretical frameworks that can engage with the multiple axes through which early motherhood is shaped and experienced best support understanding and analysis of these experiences.

In this study, intersectionality theory provided a framework whereby the complex and diverse experiences of early motherhood could be understood, as it supported analysis of the participants’ lives across multiple axes [31]. Indeed, we contend that analysis of the experiences and perspectives of young mothers must highlight the many dimensions and intersections of their lives. By using intersectionality theory, it was evident that many factors in these young mothers’ lives contributed to their early motherhood experiences. Race, age, gender, migration experience, the family environment, socioeconomic status, educational background, and social networks prior to pregnancy contributed to a complex web of intersecting experiences that then shaped teen pregnancy and early motherhood (see also [23]). Based on her research with Southeast Asian immigrant women/mothers in Australia, Liamputtong [6] argues that ‘women had other interpersonal identities that are also salient to them and impact on their mothering roles’. In this study, an array of interpersonal identities influenced how young mothers experience early motherhood. While there is a large body of literature that highlights the disadvantages of teenage pregnancy and early motherhood, intersectionality theory provides a broad framework via which the diverse contexts, experiences, drivers and outcomes of teen pregnancy and early motherhood can be considered: in this study the focus has been Sub-Saharan migrants living in Australia who experience teen pregnancy [13].

However, while acknowledging the diverse and intersecting axes that shape and define personal experiences, this paper has focused on young mother’s experiences and accounts of social support networks. The level of difficulty and regret that came with motherhood were associated with the level of social support and acceptance teenage mothers received before and after the baby’s birth. Research indicates that family support is critical to teen mothers and it has been found to have a positive impact on parenting experiences, behaviours and practices [32]. Among many participants, despite initial negative reactions – particularly from mothers - when first confronted with the news of the pregnancy, interpersonal support was forthcoming from mothers and sisters (and some female friends). Yet most spoke of a lack of support from extended family members and their own immediate communities, and this appeared to affect teen mothers’ everyday lives and futures by amplifying the disadvantages they already face. Further, the family of young mothers also came under scrutiny by the broader community, which contributed to heightened feelings of shame and disapproval. Both the fathers of the young mothers and the fathers to the babies were described as providing inadequate or even no support, often cited as being absent.

As this research found, being a teenage mother can be difficult and many participants spoke of having inadequate social support. Early motherhood was often marred by a sense of loss, particularly not being able to complete their school education and obtain a good job (see also [1, 2]). But with the right people and support, teenager mothers were able to engage in mainstream society.

Being a refugee and settling in Australia comes with many challenges, and teenage pregnancy can amplify the challenges of settlement including education, employment, housing and development of social networks [12, 33]. Yet for many participants, having a child brought a sense of purpose, family, attachment and identity. In this study, intersectionality theory has provided an important framework for examining the ‘multiple identities’ that shape pregnancy decisions and lived experiences of early motherhood among young African women living in Melbourne with refugee backgrounds [34]. For example, as young migrants from Africa with refugee backgrounds, settlement in Australia can be a highly challenging process that is often characterised by disrupted and fluid family and social networks: motherhood emerges as a lived experience that can provide stability, permanency and love. Other studies of migration and protracted refugee situations have documented that teenagers with refugee backgrounds have limited control over their lives and futures, and motherhood remains one of the few things they feel they can control [4, 19]. And in her study of South East Asian immigrants, Liamputtong [6] argues that motherhood gives young women a sense of purpose and belonging and fulfils a moral and cultural responsibility. Further, these young women come from cultures where ingrained gender roles and practices support early orientation towards motherhood and childbearing, and motherhood is regarded as a female duty and responsibility. Hence, while early motherhood has many complexities and disadvantages for young women in a site of settlement, paradoxically, it can also be fulfilling.

### Implications for service providers and policy makers

Motherhood at any age is complex, but more so for teen mothers and migrants who are developing and trying to negotiate their emerging position as adults in a site of settlement. For young women with refugee backgrounds, lack of extended family and social support networks (including the absence of biological parents) confers significant disadvantage. In this study, young mothers highlighted the important supportive role of their own mothers in particular (where available), yet many spoke of inadequate wider social support and disapproval of family and community members. Research indicates that social connectedness promotes better psychosocial outcomes for young parents, and better settlement and well-being outcomes for refugee youth [12]. Social support, both from significant people in a woman’s life and from health professionals, has been found to increase the mother’s self-confidence and assurance in her role as a mother [14, 18]. Accordingly, adequate social support for migrant/refugee teen mothers is critical, including because it has a positive impact on their ability and decisions to re-engage in education and in the workforce, thereby reducing the risk of continuing social disadvantage for mother and child. Services and teen parenting programs for young people with refugee backgrounds should recognize and facilitate the important supportive role of extended family and community networks, including mothers, siblings, guardians, friends, the father of the child, and the father (s) of the young mother.

It is important that at least one biological parent, particularly the mother, is present during and soon after the birth of the baby. This provides long-term benefits for mother and child, particularly for the mother’s re-engagement in school or work. Importantly, immigration regulations should be considered, particularly the substantial costs associated with temporary visitor visas, so that African parents/family can visit and provide much needed support for their daughters when experiencing early motherhood.

Service providers should also consider the broader context within which early, unplanned, or mistimed pregnancies and motherhood occur among teenage mothers, including those with refugee backgrounds. Despite policy commitments to delivering appropriate services to disadvantaged community, there are few examples of programs to support young mothers from refugee or culturally diverse backgrounds [12]. Initiatives and services are required that support young people to become parents while also maintaining broader settlement and life goals. Services must have increased awareness about migrant and refugee communities, and the particular challenges and needs of teen mothers, their children and families.

Given access to appropriate support, people with refugee backgrounds can make significant contributions to their new countries of settlement and lead satisfying personal and family lives. For women with refugee backgrounds, early motherhood can be challenging, particularly where there is inadequate or limited social support, and this has an impact upon their aspirations and imagined futures. Yet, in line with the emerging research that highlights the positive aspects of teen pregnancy and early motherhood, this study suggests that African young mothers with refugee backgrounds often value motherhood. Programs and policies should seek to increase and nurture social support networks while also building on the evident resilience and resourcefulness of these young women.
